# Draft Genome Sequence of Strain SA_ST125_MupR of Methicillin-Resistant *Staphylococcus aureus* ST125, a Major Clone in Spain

**DOI:** 10.1128/genomeA.00588-13

**Published:** 2013-08-08

**Authors:** Laura Barrado, Esther Viedma, Ana Vindel, Joaquín R. Otero, Fernando Chaves

**Affiliations:** Servicio de Microbiología Clínica, Hospital Universitario 12 de Octubre, Madrid, Spaina; Nosocomial Infectious Diseases Laboratory, Servicio de Bacteriología, Instituto de Salud Carlos III, Centro Nacional de Microbiología, Majadahonda, Madrid, Spainb

## Abstract

Here, we report the draft genome sequence of a methicillin-resistant *Staphylococcus aureus* (MRSA) strain with high-level mupirocin resistance (SA_ST125_MupR), isolated from a patient with recurrent bacteremia. This strain belonged to sequence type ST125, which was responsible for more than 50% of the health care-associated infections caused by MRSA in Spain.

## GENOME ANNOUNCEMENT

Methicillin-resistant *Staphylococcus aureus* (MRSA) is among the most frequently identified antimicrobial drug-resistant pathogens worldwide. It has been demonstrated that some lineages are ecologically highly successful and that most isolates belong to pandemic clones (1). In Spain, *S. aureus* sequence type ST125 continues to be responsible for more than half of the nosocomial MRSA infections (2). With increasing pressure to prevent MRSA infection, it is possible that there will be increased use of mupirocin for nasal decolonization of MRSA (3), which will be reflected in increased rates of resistance (4). High-level mupirocin resistance (Hi-Mup^R^) (MICs of ≥512 μg/ml) is mediated by acquisition of a novel isoleucyl-tRNA synthetase gene (*mup*A or *ileS*2) on a transferable plasmid, which could carry multiple resistance determinants for other classes of antimicrobial agents (3).

*S*. *aureus* ST125 was the most prevalent MRSA strain (83.3%) in our institution. We detected 8.8% Hi-Mup^R^ among MRSA isolates, and most of this resistance was focused on the ST125 clone (5). The *S. aureus* SA_ST125_MupR strain was isolated from an adult patient with recurrent bacteremia. This strain was characterized as pulsed-field gel electrophoresis pattern type E8, *spa* type t1399, *agr* type II, and staphylococcal cassette chromosome *mec* element (SCC*mec*) type IVc (6).

Genomic DNA was extracted from an overnight culture using the DNeasy blood and tissue kit (Qiagen) after addition of lysostaphin (100 μg/ml) and incubation at 37°C for 2 h. The *S. aureus* SA_ST125_MupR genome was sequenced by using the 454 GS-Junior platform (Roche), generating 196,055 reads, for a total of 91,441,914 bp, with 29-fold average coverage. With the Newbler Assembler v2.7 (Roche), the obtained sequences were *de novo* assembled into 49 contigs with an *N*_50_ contig size of 192,317 nucleotides and a total length of 2,852,031 bp. The average G+C content was 32.7%. Sequences were annotated using the NCBI Prokaryotic Genomes Automatic Annotation Pipeline (PGGAP) (http://www.ncbi.nlm.nih.gov/genome/annotation_prok/), yielding a total of 2,757 genes, 2,664 coding DNA sequence (CDS) genes, 26 pseudogenes, 58 tRNAs, and 9 rRNAs. Furthermore, the Rapid Annotations using Subsystems Technology (RAST) (http://rast.nmpdr.org) server (7) and Comprehensive Antibiotic Resistance Database (CARD) (http://arpcard.mcmaster.ca) (8) were used. There were genes related to antibiotic resistance and toxic compounds. These included efflux pumps (ATP-binding cassette [ABC] transporter, major facilitator superfamily, small multidrug resistance) potentially carrying resistance to a wide range of antimicrobials (fluoroquinolones, tetracyclines, and macrolides), and genes associated with specific resistance to β-lactams (*mec*A, *mec*R1, *mpr*F, *bla*Z, *bla*I, and *bla*R), aminoglycosides (phosphotransferases, adenyltransferases, and nucleotidyltransferases), macrolides/lincosamides/streptogramins (*mph*, *mac*B), fosfomycin (*fos*B), tetracycline (*tet*M), rifampin (*dna*A), glycopeptides (*ble*O), and mupirocin (*ile*S, *ile*S2), as well as resistance determinants to arsenic, cadmium, cobalt, mercury, and zinc. The *ile*S2 gene was located on a plasmid which was partially sequenced, giving a final size of ~58 kb, and it harbored other genes associated with resistance to β-lactams and macrolides. Overall, the availability of the present genome sequences facilitates further comparative genomic and bioinformatic analysis in *S. aureus* populations and may also provide insights into the genetic background for the success of this nosocomial lineage.

### Nucleotide sequence accession number.

This whole-genome shotgun project has been deposited at GenBank under the accession number ASTH00000000.
